# Rising burden of cancer and atrial fibrillation–related mortality among adults in the United States, 1999–2019

**DOI:** 10.1016/j.hroo.2025.05.006

**Published:** 2025-05-13

**Authors:** Muhammad Saad, Reyan Hussain Shaikh, Muhammad Umer Sohail, Saad Ahmed Waqas, Mian Muinuddin Jamshed, Syed Ibad Ahsan, Muhammad Sameer Arshad, Azeem Hassan, Sebastian Mactaggart, Raheel Ahmed

**Affiliations:** 1 Department of Medicine, Dow University of Health Sciences, Karachi, Pakistan; 2 Medical College, Aga Khan University, Karachi, Pakistan; 3 Baylor Scott and White Research Institute, Dallas, Texas; 4 National Heart and Lung Institute, Imperial College London, London, United Kingdom; 5 Northumbria Healthcare NHS Foundation Trust, North Shields, United Kingdom

**Keywords:** Cancer, Atrial fibrillation, CDC WONDER, Mortality trends, Disparities

## Abstract

**Background:**

Atrial fibrillation (AF) is the most common cardiac arrhythmia, whereas cancer remains the second leading cause of death in the United States. Both conditions share several risk factors and may compound mortality risk. However, national trends in AF and cancer-related mortality remain underexplored.

**Objective:**

To assess trends in mortality related to AF and cancer among US adults from 1999 to 2019, using demographic and geographic stratifications.

**Methods:**

Data were obtained from the CDC WONDER Multiple Cause of Death data set. We identified individuals aged 25 years and older with AF (I48) and cancer (C00-C97) as an underlying or contributing cause of death. Age-adjusted mortality rates (AAMRs) per 100,000 population, average annual percentage change, and annual percentage change were calculated.

**Results:**

The AAMR for cancer and AF-related deaths increased from 4.95 (95% confidence interval [CI]: 4.84, 5.05) in 1999 to 10.01 (95% CI: 9.88, 10.13) in 2019 (average annual percentage change: 3.49 [95% CI: 3.39, 3.60]). Males had higher AAMRs than females (10.24 vs 5.13). Non-Hispanic Whites had the highest AAMRs (7.89), followed by non-Hispanic Blacks (4.53) and Hispanics (3.11). The Western region had the highest AAMR, and nonmetropolitan areas exhibited greater mortality rates than metropolitan areas. Individuals with lung cancer exhibited the highest AAMRs (1.65), followed by gastrointestinal cancer (1.52) and hematologic cancer (1.00). The lowest AAMRs were observed in prostate cancer (0.90) and breast cancer (0.65).

**Conclusion:**

Mortality from cancer and AF has increased over time, with significant disparities across sex, race, and geography. Targeted interventions are required to mitigate these disparities.

## Graphical abstract

**Figure:**
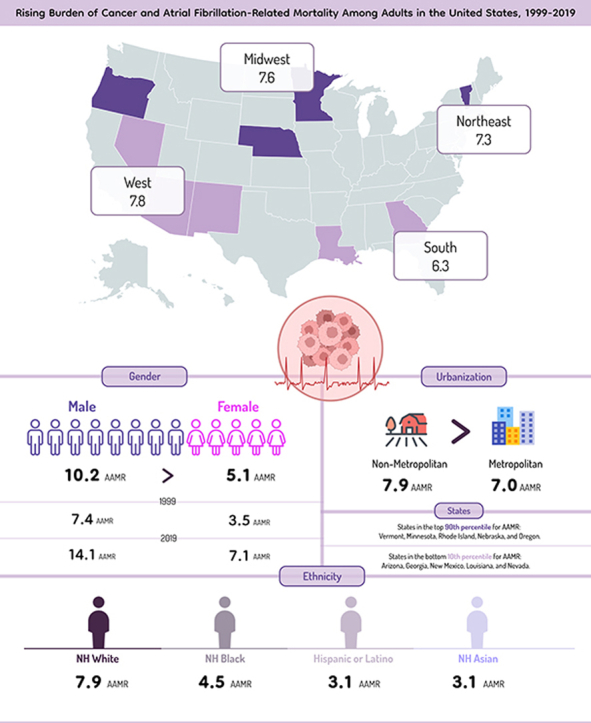

**Key Findings**

▪ Rising mortality rates: The age-adjusted mortality rate (AAMR) for cancer and atrial fibrillation-related deaths increased from 4.95 (95% confidence interval [CI]: 4.84–5.05) in 1999 to 10.01 (95% CI: 9.88–10.13) in 2019, with an average annual percentage change of 3.49% (95% CI: 3.39–3.60).
▪ Sex disparities: Men exhibited higher AAMRs than women (10.24 vs 5.13). Both sexes experienced increasing trends, with men having an average annual percentage change of 3.30% and women having 3.36%.
▪ Racial disparities: Non-Hispanic (NH) White individuals had the highest AAMRs (7.89), followed by NH Black (4.53), Hispanic (3.11), and NH Asian or Pacific Islander individuals (3.07).
▪ Geographic disparities: The Western region exhibited the highest AAMRs, whereas nonmetropolitan areas consistently had greater mortality rates than metropolitan areas.
▪ Cancer subtypes: Individuals with lung cancer exhibited the highest AAMRs (1.65), followed by gastrointestinal cancer (1.52) and hematologic cancer (1.00). The lowest AAMRs were observed in prostate cancer (0.90) and breast cancer (0.65).

## Introduction

Atrial fibrillation (AF) is the most common cardiac arrhythmia that is clinically observed and is associated with significant morbidity and mortality globally.^1^ Its prevalence is increasing globally due to several risk factors, including increasing age, hypertension, obesity, diabetes, and smoking.^1^^,^^2^ Furthermore, cancer is the second most common cause of death in the United States and shares several risk factors with AF.^3^ Although cancer mortality has declined in the past 3 decades, projections estimate >2 million new cancer cases and approximately 600,000 associated deaths in 2024, alongside a rising AF prevalence expected to affect 12.1 million Americans by 2030.^4^^,^^5^

Several studies have documented that a bidirectional relationship exists between these conditions, with patients with cancer experiencing a heightened risk of AF due to autonomic dysfunction, structural and electrical remodelling of the heart, and homeostasis-related ion abnormalities.^6^ These abnormalities are caused by several factors, such as proinflammatory immune activation, postsurgical inflammatory responses, oxidative stress, chronic obstructive pulmonary disease, and cardiotoxic effects of chemotherapy and radiotherapy.^6^ Studies suggest a 47% increased AF risk in patients with cancer overall and a 20% increased risk in those not actively undergoing cancer treatment.^7^^,^^8^ Conversely, AF may elevate cancer risk by disrupting apoptosis, potentially favoring carcinogenesis. The co-occurrence of AF and cancer compounds mortality risk, increasing the likelihood of thromboembolism and a 6-fold increased risk of heart failure.^9^

Despite well-documented independent mortality trends for cancer and AF, trends in mortality among individuals with cancer and AF, specifically across racial, socioeconomic, and geographic groups, remain unknown. To address this gap, we analyzed Centers for Disease Control and Prevention’s Wide-ranging Online Data for Epidemiologic Research (CDC WONDER) data set to evaluate trends in cancer and AF-related mortality in the United States from 1999 to 2019, stratified by sex, race or ethnicity, states, census regions, and urbanization.

## Methodology

### Study setting and population

The CDC WONDER Multiple Cause of Death data set was used for death certificate records that listed cancer and AF were recorded as a contributing or underlying cause of death, during 1999 to 2019.^10^ The study focused on individuals aged 25 years and older, identified by the International Classification of Diseases, Tenth Revision codes I48 for AF and C00-C97 for cancer.^11^, ^12^, ^13^ Because the data were deidentified and publicly available, our study was exempt from local institutional review board approval and follows the Strengthening the Reporting of Observational Studies in Epidemiology guidelines for reporting.

### Data abstraction

The data were grouped by demographic variables, including sex, race, or ethnicity, and geographical variables, such as state wise, census regions, and urbanization. Racial or ethnic groups were defined as Hispanic or Latino, non-Hispanic (NH) White, NH Black or African American, NH American Indian or Alaskan Native, and NH Asian. The National Center for Health Statistics’ Urban-Rural Classification Scheme was used to categorize mortality trends by urbanization status. Small to medium metropolitan areas (population 50,000–999,000) and large metropolitan cities (populations >1 million) are classified as urban areas, whereas rural areas comprise smaller locales with populations <50,000. In accordance with the US Census Bureau’s classification, we categorized regions into Northeast, Midwest, South, and West.^14^ To investigate differences in AF-related mortality among various cancer subtypes, we evaluated the following 5 prevalent cancer types: lung (C34), gastrointestinal (C15–C26), prostate (C61), breast (C50), and hematologic malignancies (C81–C96). These cancer sites were selected based on national statistics, representing the most frequently diagnosed cancers.^4^

### Statistical analysis

To investigate national trends in AF and cancer-related mortality, we calculated crude and age-adjusted mortality rates (AAMRs) per 100,000 individuals over the study period. Crude mortality rates were obtained by dividing the annual number of deaths by the respective US population for each year, whereas AAMRs were standardized to the 2000 US population for consistency across years.^15^ The 2000 US census data formed the standard population for calculating AAMRs. We reported 95% CIs for all mortality rates. Data for NH American Indian or Alaskan Native populations were excluded from trend analyses due to unreliable reporting. Trend analyses were conducted using the Joinpoint Regression Program (version 5.2.0, National Cancer Institute), which calculates annual percent change (APC) and average annual percent change (AAPC) along with corresponding 95% CIs. This involved fitting log-linear regression models in which temporal changes in trends occurred, to evaluate statistically significant variations in AAMR over a time period. A trend was classified as increasing or decreasing if its slope significantly differed from 0, with statistical significance assessed using 2-tailed *t* tests. A *P* value ≤ .05 was considered statistically significant. Last, a parallelism test was performed to evaluate whether the trend in AAMRs for cancer-related mortality differed significantly from the trend in AAMRs for cancer and AF-related mortality and whether the trend in AAMRs for AF-related mortality differed significantly from the trend in AAMRs for cancer and AF-related mortality. A significant *P* value in this interaction test confirmed that the 2 trends, when assessed in terms of AAPC, were statistically distinct from each other.

## Results

A total of 319,480 cancer and AF-related deaths occurred among adults (aged 25 years and older) between 1999 and 2019. The summarized results of AAMRs per 100,000, categorized by variables annual, gender, and race are illustrated in the Central Illustration.

### Annual trends for cancer and AF-related AAMR

In 1999, the AAMR for cancer and AF-related deaths in adults was 4.95 (95% CI: 4.84, 5.05), increasing to 10.01 (95% CI: 9.88, 10.13) in 2019, with an AAPC of 3.49 (95% CI: 3.39, 3.60). The AAMR rose from 1999 to 2015 (APC: 3.03 [95% CI: 2.87, 3.17]) and surged until 2019 (APC: 5.36 [95% CI: 4.63, 6.62]).

**Figure 1:**
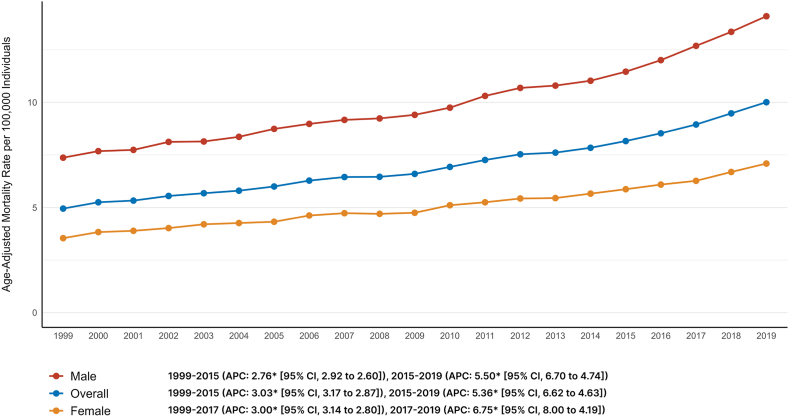
Trends in overall and sex-stratified age-adjusted cancer and AF-related mortality in the United States from 1999 to 2019. AF = atrial fibrillation; APC = annual percent change; CI = confidence interval.

Visual trends are illustrated in Figure 1 with comprehensive data present in Supplemental Table 1.

### Cancer and AF-related AAMR stratified by sex

Throughout the study period, males consistently exhibited higher AAMRs compared with females (AAMR: 10.24 [95% CI: 10.19, 10.29] vs 5.13 [95% CI: 5.10, 5.16]). However, both genders experienced a similar increase in AAMRs (males: AAPC: 3.30 [95% CI: 3.20, 3.42]; females: AAPC: 3.36 [95% CI: 3.18, 3.50]; Figure 1).

Visual trends are illustrated in Figure 1 with comprehensive data present in Supplemental Table 1.

### Cancer and AF-related AAMR stratified by race

**Figure 2:**
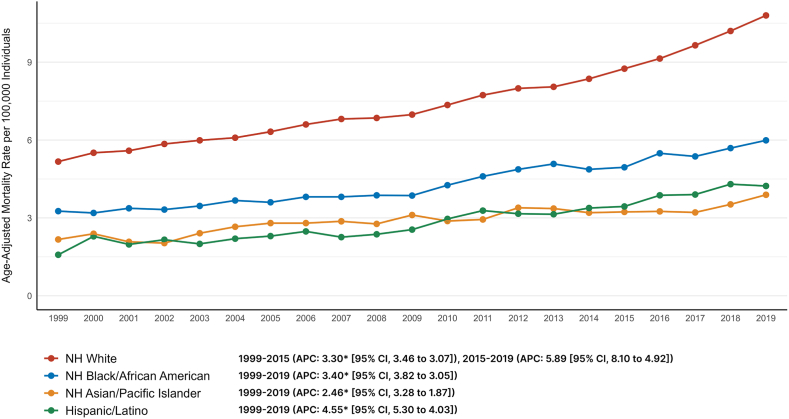
Trends in age-adjusted cancer and AF-related mortality in the United States stratified by race or ethnicity from 1999 to 2019. AF = atrial fibrillation; APC = annual percent change; CI – confidence interval; NH = non-Hispanic.

Among racial groups, the NH White population demonstrated the highest AAMR at 7.89 (95% CI: 7.86, 7.92), followed by NH Black at 4.53 (95% CI: 4.46, 4.60), Hispanic or Latino at 3.11 (95% CI: 3.04, 3.17), and NH Asian or Pacific Islander at 3.07 (95% CI: 2.98, 3.16). The trends for NH White individuals had a steady increase from 1999 to 2015 (APC: 3.30 [95% CI: 3.07, 3.46]), followed by a sharp rise until 2019 (APC: 5.89 [95% CI: 4.92, 8.10]). For NH Black or African Americans, there was a consistent increase from 1999 to 2019 (APC: 3.40 [95% CI: 3.05, 3.82]). For Hispanic or Latinos, there was a sharp rise throughout the study duration (APC: 4.55 [95% CI: 4.03, 5.30]). For NH Asian or Pacific Islander individuals, there was a rise from 1999 to 2019 (APC: 2.46 [95% CI: 1.87, 3.28]). Hispanics had the greatest AAMR increase (AAPC: 4.55; [4.03–5.30]), followed by NH Whites (AAPC: 3.81; [3.67–3.93]), NH Blacks (AAPC: 3.40; [3.05–3.82]), and NH Asian or Pacific Islanders (AAPC: 2.46; [1.87–3.28]) (Figure 2).

Visual trends are illustrated in Figure 2 with comprehensive data present in Supplemental Table 2.

### Cancer and AF-related AAMR stratified by geographic region

#### State and census-wise analysis

Significant disparities in AAMRs across states were observed from 1999 to 2019. States such as Oregon (AAMR: 10.7 [95% CI: 10.4, 10.9]), Nebraska (AAMR: 10.7 [95% CI: 10.3, 11.1]), Minnesota (AAMR: 11.1 [95% CI: 10.9, 11.3]), Vermont (AAMR: 11.9 [95% CI: 11.3, 12.6]), and Rhode Island (AAMR: 11.1 [95% CI: 10.6, 11.6]) ranked in the top 90th percentile. Nevertheless, states such as Arizona (AAMR: 4.3 [95% CI: 4.2, 4.4]), Georgia (AAMR: 4.3 [95% CI: 4.1, 4.4]), New Mexico (AAMR: 4.3 [95% CI: 4, 4.5]), Louisiana (AAMR: 3.9 [95% CI: 3.7, 4.1]), and Nevada (AAMR: 3.4 [95% CI: 3.2, 3.6]) ranked in the bottom 10th percentile.

From 1999 to 2019, the highest overall AAMR was recorded in the West Region (AAMR: 7.8 [95% CI: 7.74, 7.85]), followed by the Midwest (AAMR: 7.64 [95% CI: 7.58, 7.69]), the Northeast (AAMR: 7.32 [95% CI: 7.26, 7.38]), and the South (AAMR: 6.34 [95% CI: 6.3, 6.38]). The South experienced the steepest rise in AAMRs between 2015 and 2019 (APC: 7.97 [95% CI: 6.50, 11.10]), whereas the Northeast saw the least increase between 1999 and 2008 (APC: 1.28 [95% CI: 0.27, 1.77]). The overall AAPC for the Western region was +3.5 percent per year (95% CI: +3.3, +3.7). For Midwest regions, the AAPC increased by +3.5 percent per year (95% CI: +3.4, +3.7).

**Figure 3:**
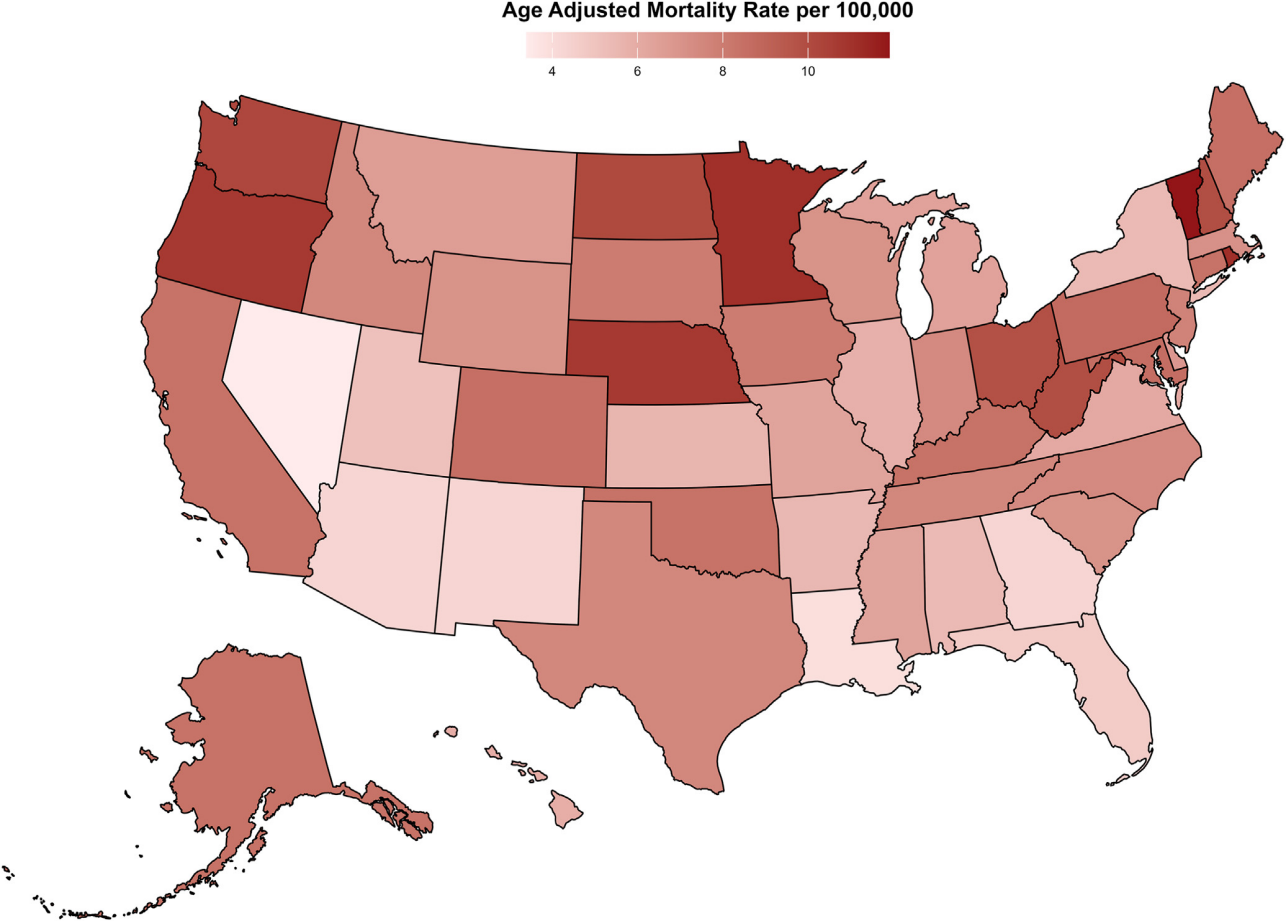
Trends in age-adjusted cancer and AF-related mortality in the United States stratified by state from 1999 to 2019. AF = atrial fibrillation.

A state-wise map highlighting regional disparities and visual trends is presented in Figure 3, with comprehensive data available in Supplemental Table 3 and Supplemental Table 4.

#### Urbanization-wise analysis

The AAMRs for non-metropolitan areas (AAMR: 7.89, [95% CI: 7.83, 7.96]) were consistently higher than those for metropolitan areas (AAMR: 7, [95% CI: 6.98, 7.03]). For nonmetropolitan areas, there was a steady increase in AAMRs from 1999 to 2014 (APC: 3.31, [95% CI: 2.87, 3.67]), followed by a sharp rise until 2019 (APC: 6.80, [95% CI: 5.57, 9.20]). The AAPC in nonmetropolitan areas had an increase of +4.2 percent per year (95% CI: +3.9, +4.4). For metropolitan areas, there was a consistent increase from 1999 to 2015 in AAMRs (APC: 2.94, [95% CI: 2.73, 3.11]) and from 2015 to 2019 (APC: 4.87, [95% CI: 3.94, 6.92]). The AAPC in metropolitan areas had an increase of +3.3 percent per year (95% CI: +3.2, +3.5).

**Figure 4:**
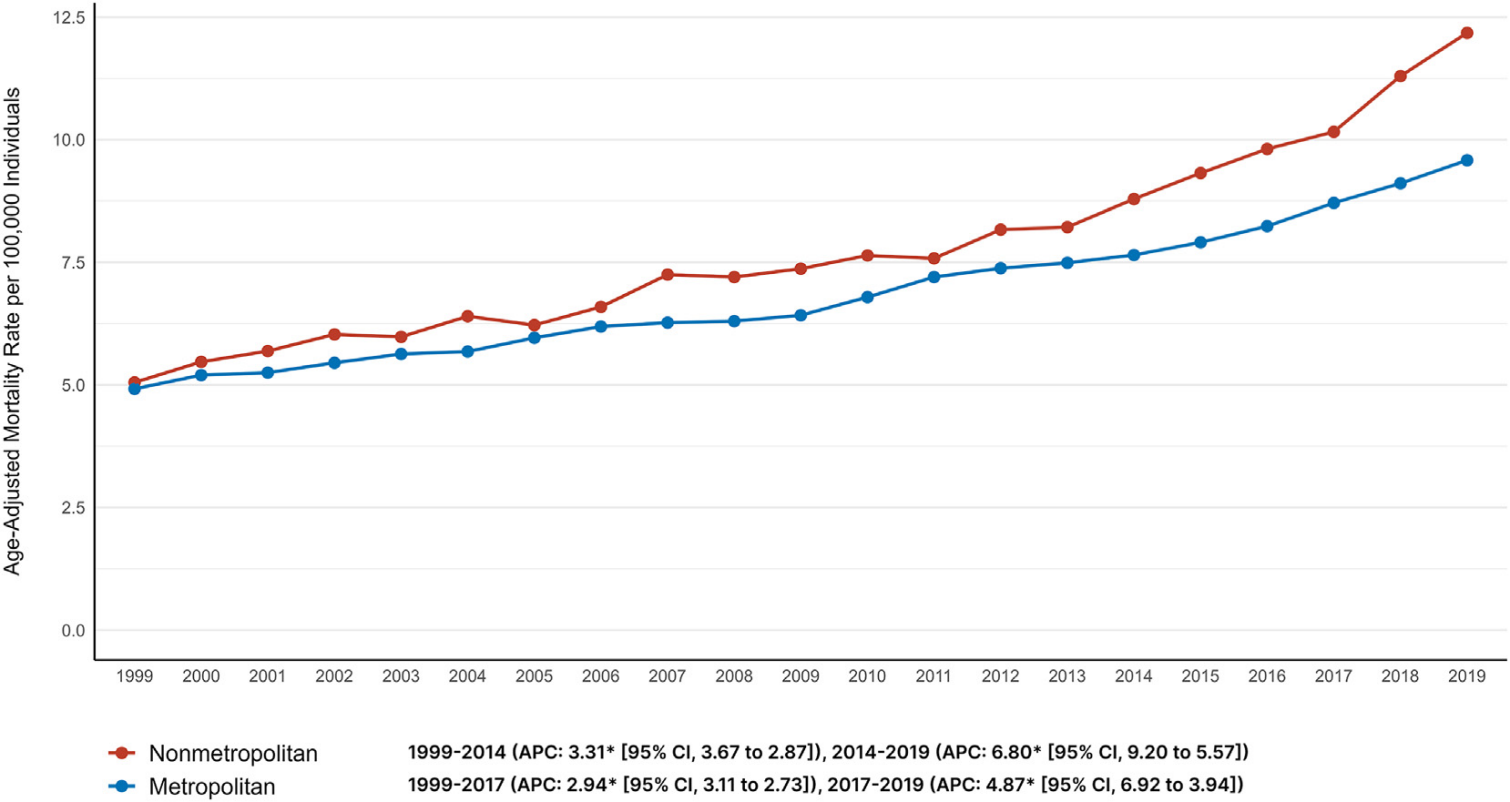
Trends in age-adjusted cancer and AF-related mortality in the United States stratified by the level of urbanization from 1999 to 2019. AF = atrial fibrillation.

Visual trends are illustrated in Figure 4 with comprehensive data present in Supplemental Table 5.

### Annual trends for cancer-related AAMR

There were a total of 13,197,629 deaths due to cancer alone in the period 1999 to 2019. In 1999, the AAMR for cancer-related deaths in adults was 343.73 (95% CI: 342.87, 344.6), which decreased to 249.2 (95% CI: 248.59, 249.81) in 2019. From 1999 to 2019, there was a steady decline (APC: −1.61 [95% CI: −1.63, −1.58]). On sensitivity analysis, AAMR for cancer alone as underlying or contributing cause of death had a declining trend (p for parallelism <0.001), indicating that the trend for cancer-related AAMR was significantly different from the overall trend of cancer- and AF-related AAMR.

Visual trends with APCs are illustrated in Supplemental Figure 1 and Supplemental Table 6.

### Annual trends for AF-related AAMR

There were a total of 2,365,381 deaths due to AF alone in the period 1999 to 2019. In 1999, the AAMR for AF-related deaths in adults was 38.16 (95% CI: 37.87, 38.45), which increased to 69.32 (95% CI: 69, 69.64) in 2019. From 1999 to 2009, there was a steady increase (APC: 2.29 [95% CI: 1.15, 2.76]), and this was followed by a steeper increase from 2009 to 2019 (APC: 3.68 [95% CI: 3.34, 4.59]). On sensitivity analysis, AAMR for AF alone as the underlying or contributing cause of death had an increasing trend (p for parallelism >0.001), indicating that the trend for AF-related AAMR was significantly different from the overall trend of cancer- and AF-related AAMR.

Visual trends with APCs are illustrated in Supplemental Figure 1 and Supplemental Table 7.

### Cancer subtypes

The highest overall AAMR across different types of cancer were found with lung cancer 1.65 (95% CI: 1.64, 1.66), followed by gastrointestinal cancer 1.52 (95% CI: 1.51, 1.53) and hematologic cancer 1.00 (95% CI: 0.99, 1.01). The lowest AAMRs were observed in prostate cancer at 0.90 (95% CI: 0.89, 0.91) and breast cancer at 0.65 (95% CI: 0.64, 0.65). The various types of cancer associated with AF exhibited an overall increasing trend from 1999 to 2019, where the greatest increase was observed in hematologic cancer (AAPC: 4.91 [95% CI: 4.55, 5.37]), followed by lung cancer (AAPC: 3.28 [95% CI: 3.12, 3.47]), breast cancer (AAPC: 2.87 [95% CI: 2.49, 3.16]), prostate cancer (AAPC: 2.85 [95% CI: 2.69, 3.08]), and gastrointestinal cancer (AAPC: 2.83 [95% CI: 2.64, 3.02]).

Visual trends with APCs are illustrated in Supplemental Figure 2 and Supplemental Table 8.

## Discussion

Our analysis reveals a significant and sustained increase in cancer- and AF-related mortality among US adults in the past 2 decades. Our results indicate a rise in overall cancer- and AF-related mortality, which was significantly different from the overall trend in cancer-related mortality and AF-related mortality. A similar pattern of rising mortality was observed across both sexes and all racial groups. Males consistently exhibited higher mortality compared with females throughout the study period, with NH White Americans experiencing the highest mortality burden among all racial groups. Non-metropolitan areas had a greater burden of mortality compared with metropolitan areas. Significant geographic disparities persist, with the highest mortality observed in the Western and Midwestern regions, followed by the Northeast, and the lowest mortality in the South. States in the top 90th percentile had more than 2.5 times the AAMRs compared with states in the bottom 10th percentile. The highest overall mortality burden was found with lung cancer, followed by gastrointestinal and hematologic cancers, whereas breast and prostate cancers contributed comparatively lower mortality.

Our findings align with the previous literature, particularly a brief report uncovering the trends in cardiovascular mortality among patients with AF and cancer in United States.^16^ However, unlike the previously published brief report, which stratified mortality trends in AF and patients with cancer only by combined race-sex groups, our study offers greater granularity by independently analyzing trends by sex and race. We also uniquely evaluate geographic disparities across census regions, states, and urban-rural settings—areas not addressed in the previous study. In addition, our analysis disaggregates mortality by cancer subtypes, providing insights into subtype-specific trends. The previous paper also primarily revolves around underlying cardiovascular mortality, leading to exclusion of non–cardiovascular-related deaths. Together, these methodological enhancements offer a more comprehensive and nuanced understanding of AF and cancer-related mortality in United States.

The bidirectional association between cancer and AF is well documented.^17^ The advancements in cancer treatment, along with early detection, have markedly improved the cancer outcomes and survival rates.^18^ Despite this, patients with cancer remain at an increased risk of cardiotoxic effects due to treatment regimens known to affect cardiac function.^19^ Several studies have corroborated this risk. A study of >3 million patients with cancer with concomitant cardiac disease reported an increase in mortality between 1973 and 2012.^20^ Specifically for AF, patients with cancer have an incidence as high as 30%, compared with approximately 2% in the general population.^3^ A meta-analysis further highlighted that patients with cancer have a 47% greater risk of developing AF than those without cancer, placing them at an elevated risk of mortality.^21^^,^^22^ Many anticancer treatment therapies have been found to contribute to the development of AF, including tyrosine kinase inhibitors, taxanes, antimetabolites, alkylating agents such as cyclophosphamide and cisplatin, and topoisomerase II inhibitors.^6^^,^^23^ In addition to treatment-related cardiotoxicity, postoperative AF is another factor that may worsen outcomes in patients with cancer.^3^

Our results indicate a consistent decline in overall cancer-related mortality over the years, whereas AF-related mortality has demonstrated an upward trajectory. This suggests that the rising burden of AF-related mortality may be contributing to the overall increase in combined cancer- and AF-related mortality. Notably, AF-related mortality has been rising since 1980,^24^ likely driven by an increasing prevalence of risk factors, such as hypertension, diabetes, and obesity, particularly among younger adults.^25^, ^26^, ^27^ However, older adults also remain at heightened risk, given the greater prevalence of permanent or persistent AF with increasing age. The elevated mortality in older adults may also be driven by concurrent comorbidities associated with aging.^28^

Although similar reasons explain the rising burden of cancer- and AF-related mortality among both sexes, it is pertinent to note that males consistently exhibited a higher AAMR as compared with females. This is strange because more females are likely to die from AF as compared with males, as has been found by many studies.^29^^,^^30^ This indicates that the disparities observed in sex are largely driven by cancer-related mortality, as males are at an increased risk of mortality as compared with females across many cancer types.^31^ In addition, the cardiotoxicity associated with the anthracycline treatment is more likely to yield worse adverse effects in male adults as compared with female adults.^32^ Although the exact reasons for a protective effect in females need to be elucidated, it is believed that the female hormones play a role in reducing oxidative stress on heart.^33^^,^^34^ Despite this, there is a paucity of literature on sex-based disparities in cancer- and AF-related mortality. It is worth adding that immune-related adverse events due to immunotherapy, including cardiotoxicity associated with radiation therapy, are more likely to occur in females than males.^32^ Therefore, we implore that further studies should be conducted to explore this specific sex disparity observed in our study and elucidate the reasons contributing to it.

NH White Americans had the greatest burden of mortality from cancer- and AF-related mortality as compared with other races and ethnic groups.^35^ Interestingly, the risk factors associated with AF, including hypertension, diabetes, and obesity, are greater in NH Blacks as compared with NH Whites.^36^, ^37^, ^38^ Similarly, coronary artery disease and diabetes are more prevalent in American Indians as compared with other races.^39^^,^^40^ A cohort study found that Black and Hispanic patients were 23% and 13% less likely to initiate direct oral anticoagulants, respectively, compared with White patients.^41^ Therefore, despite the higher prevalence of risk factors and treatment disparities in other races, the NH Whites are still predisposed to higher prevalence and mortality from AF—this is referred to as “racial paradox.”^41^ These disparities are influenced by several factors. For instance, it is probable that there is underdiagnosis of AF in Blacks and American Indians, along with the possibility of limited access to routine electrocardiogram screening that could detect AF. What further adds to this paradox is that the Blacks and American Indians have greater mortality due to cancer as compared with the Whites, with the highest mortality burden found in Blacks in 2019 in United States.^42^^,^^43^ Despite this, in our study, the mortality is highest among the Whites due to both cancer and AF. However, because the mortality trends in our study largely mirror the disparities observed among racial groups in AF, it may be AF-related prevalence and mortality that is largely driving the mortality in our study.

Our study revealed that the mortality was consistently higher in non-metropolitan regions as compared with metropolitan regions. The lower socioeconomic status and higher poverty in rural areas explain this disparity. Rural areas have been found to have a higher burden of mortality in patients with comorbid cancer and cardiovascular diseases in many studies.^44^^,^^45^ Socioeconomic disparities have been found to adversely affect cancer and cardiovascular outcomes, primarily due to poor access to good-quality health care.^46^^,^^47^ In addition, rural areas also lack substantial tertiary care setups, with limited cardio-oncology specialists.^48^^,^^49^ A previous study revealed that those patients with cancer residing in neighborhoods with lower socioeconomic standing have a greater risk of developing cardiac disease as compared with those living in higher socioeconomic settings.^50^ Another large Surveillance, Epidemiology, and End Results registry-based study revealed that the risk of cardiovascular disease mortality among patients with cancer increased with worsening poverty.^51^ This elevated risk persisted even after adjusting for race and ethnicity. Poor income also increases the likelihood of being uninsured, which in turn worsens the outcomes. A study revealed that patients with cancer who had Medicaid insurance had lower cardiovascular mortality as compared with those who did not have insurance.^52^

Significant geographic disparities exist, with the highest mortality rates being observed in the West (7.8) and Midwest (7.64) and the lowest rates being found in the South (6.34). These substantial regional differences can be attributed to multiple factors, including disparities in the use of evidence-based medications, variations in ambulatory cardiology practices, barriers to accessing quality health care, and the influence of state Medicaid policies.^53^ The disparities observed among states require further investigation, as the current data are limited. Vermont, which had the highest AAMR, also has the largest rural population, suggesting the low socioeconomic status may be driving the mortality rates.^54^ However, this pattern does not consistently apply to the other states in the top 90th percentile, indicating that additional factors may be contributing to these variations and warranting further research.

Previous studies have revealed that the risk of developing AF is particularly elevated in several cancer subtypes, including lung, gastrointestinal, colorectal, hematologic, and genitourinary malignancies.^55^, ^56^, ^57^, ^58^ This could be due to nonspecific shared risk factors, such as systemic inflammation as signified by elevated C-reactive protein levels, and an imbalance between sympathetic and parasympathetic systems causing autonomic dysfunction in AF.^59^^,^^60^ In particular, the highest mortality in our study was observed in lung cancer. It is noteworthy that most AF cases in lung patients with cancer are reported post-surgery, likely related to the complications associated with medical and surgical treatment apart from the direct effects of the malignancy itself.^61^

Future research should aim to clarify the temporal dynamics and causal pathways linking AF and cancer, particularly whether mortality risk differs based on the sequence of disease onset. Longitudinal, patient-level data sets, including cancer registries, electronic health records, and insurance claims, will be instrumental in distinguishing new-onset AF in patients with cancer from pre-existing comorbidity and in assessing treatment exposures such as chemotherapy, radiation, anticoagulation, and AF ablation. Integration of procedural and pharmacologic data could contextualize mortality trends and inform targeted interventions. In addition, linking clinical outcomes with social determinants of health, including insurance coverage, socioeconomic status, and geographic access to care, may help explain disparities observed across racial, regional, and urban-rural lines. Investigating provider-level and system-level factors such as cardio-oncology specialist availability and infrastructure may also be valuable. Finally, prospective studies evaluating early AF screening in oncology populations and implementation of integrated cardio-oncology care models, particularly in high-risk or underserved regions, could help reduce excess mortality in this growing dual-disease population.

This study has several limitations. First, the use of International Classification of Diseases–coded death certificate data may result in misclassification of cause of death. The CDC WONDER data set lacks clinical detail, including disease chronology, cancer stage, treatment modalities, and medical therapy, precluding causal inference and distinction between new-onset vs pre-existing AF. Differences in diagnostic practices or coding trends over time and across regions may have influenced AF documentation. In addition, social determinants of health such as income, insurance status, and access to palliative or specialty care could not be assessed, limiting our ability to explain disparities in rural and minority populations. Despite these limitations, the data set’s large, nationally representative scope allows for robust assessment of population-level trends and disparities that smaller clinical data sets may not capture.

## Conclusion

In conclusion, our study reveals a sustained rise in cancer- and AF-related mortality, with significant disparities across sex, race, and geography. The increasing AF-related mortality highlights the need for better AF screening and cardiotoxicity management in patients with cancer. Higher mortality in males and NH Whites, along with the observed geographic variations warrant further investigations. Higher mortality in rural areas underscores health care access disparities. Addressing these disparities through targeted interventions, improved cardio-oncology care, and equitable health care access is crucial.

## Disclosures

The authors have no conflicts of interest to declare.
