# Supplementary material for: Rising burden of cancer and atrial fibrillation–related mortality among adults in the United States, 1999–2019
Source: Heart Rhythm O2. 2025 May 13;6(8):1130–8. doi: 10.1016/j.hroo.2025.05.006 (PMC12411967; doi:10.1016/j.hroo.2025.05.006)

**Supplementary Table 1.** Cancer and AF–related age-adjusted mortality rates per 100,000, stratified by sex in adults in the United States, 1999 to 2019.

| **Year** | **Sex** | **Deaths** | **Population** | **Age Adjusted Rate** | **Age Adjusted Rate Lower 95% Confidence Interval** | **Age Adjusted Rate Upper 95% Confidence Interval** |
| --- | --- | --- | --- | --- | --- | --- |
| **1999** | **Female** | 3931 | 94123092 | 3.54 | 3.43 | 3.65 |
| **2000** | **Female** | 4315 | 94864102 | 3.83 | 3.72 | 3.94 |
| **2001** | **Female** | 4451 | 95984408 | 3.89 | 3.78 | 4.01 |
| **2002** | **Female** | 4622 | 96927703 | 4.02 | 3.9 | 4.14 |
| **2003** | **Female** | 4879 | 97893297 | 4.2 | 4.08 | 4.32 |
| **2004** | **Female** | 4990 | 98921524 | 4.26 | 4.14 | 4.38 |
| **2005** | **Female** | 5165 | 100097839 | 4.32 | 4.2 | 4.44 |
| **2006** | **Female** | 5545 | 101328442 | 4.62 | 4.49 | 4.74 |
| **2007** | **Female** | 5782 | 102513830 | 4.73 | 4.61 | 4.86 |
| **2008** | **Female** | 5841 | 103688156 | 4.7 | 4.58 | 4.82 |
| **2009** | **Female** | 6009 | 104834186 | 4.75 | 4.63 | 4.87 |
| **2010** | **Female** | 6553 | 105717426 | 5.11 | 4.99 | 5.24 |
| **2011** | **Female** | 6874 | 107004480 | 5.25 | 5.12 | 5.38 |
| **2012** | **Female** | 7223 | 108088829 | 5.43 | 5.3 | 5.55 |
| **2013** | **Female** | 7408 | 109172782 | 5.45 | 5.32 | 5.57 |
| **2014** | **Female** | 7831 | 110581619 | 5.66 | 5.53 | 5.79 |
| **2015** | **Female** | 8287 | 111947362 | 5.87 | 5.74 | 6 |
| **2016** | **Female** | 8751 | 112990913 | 6.09 | 5.96 | 6.22 |
| **2017** | **Female** | 9278 | 114358026 | 6.27 | 6.14 | 6.4 |
| **2018** | **Female** | 10084 | 115265998 | 6.69 | 6.56 | 6.83 |
| **2019** | **Female** | 10874 | 116084432 | 7.09 | 6.95 | 7.22 |
| **1999** | **Male** | 4790 | 86285677 | 7.37 | 7.16 | 7.58 |
| **2000** | **Male** | 5060 | 87120538 | 7.68 | 7.46 | 7.9 |
| **2001** | **Male** | 5210 | 88320720 | 7.74 | 7.53 | 7.96 |
| **2002** | **Male** | 5562 | 89280325 | 8.12 | 7.9 | 8.34 |
| **2003** | **Male** | 5720 | 90197132 | 8.14 | 7.93 | 8.36 |
| **2004** | **Male** | 5987 | 91283860 | 8.36 | 8.14 | 8.57 |
| **2005** | **Male** | 6393 | 92453545 | 8.74 | 8.52 | 8.95 |
| **2006** | **Male** | 6745 | 93690917 | 8.98 | 8.76 | 9.19 |
| **2007** | **Male** | 7092 | 94889947 | 9.17 | 8.96 | 9.39 |
| **2008** | **Male** | 7324 | 96106934 | 9.24 | 9.02 | 9.45 |
| **2009** | **Male** | 7667 | 97272830 | 9.41 | 9.2 | 9.62 |
| **2010** | **Male** | 8098 | 98174557 | 9.75 | 9.53 | 9.96 |
| **2011** | **Male** | 8879 | 99588456 | 10.31 | 10.09 | 10.53 |
| **2012** | **Male** | 9489 | 100737208 | 10.69 | 10.48 | 10.91 |
| **2013** | **Male** | 9927 | 101912532 | 10.8 | 10.59 | 11.02 |
| **2014** | **Male** | 10478 | 103227661 | 11.03 | 10.81 | 11.24 |
| **2015** | **Male** | 11203 | 104606455 | 11.46 | 11.25 | 11.68 |
| **2016** | **Male** | 12098 | 105650504 | 12.01 | 11.79 | 12.22 |
| **2017** | **Male** | 13191 | 107089305 | 12.69 | 12.47 | 12.91 |
| **2018** | **Male** | 14315 | 108045192 | 13.36 | 13.14 | 13.58 |
| **2019** | **Male** | 15559 | 108896735 | 14.1 | 13.87 | 14.32 |
| **1999** | **Overall** | 8721 | 180408769 | 4.95 | 4.84 | 5.05 |
| **2000** | **Overall** | 9375 | 181984640 | 5.25 | 5.14 | 5.36 |
| **2001** | **Overall** | 9661 | 184305128 | 5.33 | 5.22 | 5.44 |
| **2002** | **Overall** | 10184 | 186208028 | 5.55 | 5.44 | 5.65 |
| **2003** | **Overall** | 10599 | 188090429 | 5.68 | 5.58 | 5.79 |
| **2004** | **Overall** | 10977 | 190205384 | 5.8 | 5.69 | 5.91 |
| **2005** | **Overall** | 11558 | 192551384 | 6 | 5.89 | 6.11 |
| **2006** | **Overall** | 12290 | 195019359 | 6.28 | 6.17 | 6.39 |
| **2007** | **Overall** | 12874 | 197403777 | 6.45 | 6.33 | 6.56 |
| **2008** | **Overall** | 13165 | 199795090 | 6.46 | 6.35 | 6.57 |
| **2009** | **Overall** | 13676 | 202107016 | 6.6 | 6.49 | 6.71 |
| **2010** | **Overall** | 14651 | 203891983 | 6.93 | 6.82 | 7.04 |
| **2011** | **Overall** | 15753 | 206592936 | 7.26 | 7.14 | 7.37 |
| **2012** | **Overall** | 16712 | 208826037 | 7.53 | 7.41 | 7.64 |
| **2013** | **Overall** | 17335 | 211085314 | 7.61 | 7.49 | 7.72 |
| **2014** | **Overall** | 18309 | 213809280 | 7.84 | 7.72 | 7.95 |
| **2015** | **Overall** | 19490 | 216553817 | 8.16 | 8.04 | 8.27 |
| **2016** | **Overall** | 20849 | 218641417 | 8.53 | 8.41 | 8.64 |
| **2017** | **Overall** | 22469 | 221447331 | 8.95 | 8.83 | 9.07 |
| **2018** | **Overall** | 24399 | 223311190 | 9.48 | 9.36 | 9.6 |
| **2019** | **Overall** | 26433 | 224981167 | 10.01 | 9.88 | 10.13 |

**Supplementary Table 2.** Cancer and AF–related age-adjusted mortality rates per 100,000, stratified by race in adults in the United States, 1999 to 2019.

| **Year** | **Race** | **Deaths** | **Population** | **Age Adjusted Rate** | **Age Adjusted Rate Lower 95% Confidence Interval** | **Age Adjusted Rate Upper 95% Confidence Interval** |
| --- | --- | --- | --- | --- | --- | --- |
| **1999** | **NH Asian or Pacific Islander** | 76 | 6917321 | 2.13 | 1.66 | 2.68 |
| **2000** | **NH Asian or Pacific Islander** | 87 | 7210256 | 2.38 | 1.89 | 2.95 |
| **2001** | **NH Asian or Pacific Islander** | 87 | 7651274 | 2.1 | 1.67 | 2.61 |
| **2002** | **NH Asian or Pacific Islander** | 87 | 7999855 | 1.99 | 1.58 | 2.46 |
| **2003** | **NH Asian or Pacific Islander** | 108 | 8336941 | 2.44 | 1.97 | 2.91 |
| **2004** | **NH Asian or Pacific Islander** | 131 | 8679634 | 2.67 | 2.2 | 3.13 |
| **2005** | **NH Asian or Pacific Islander** | 145 | 9041913 | 2.78 | 2.32 | 3.25 |
| **2006** | **NH Asian or Pacific Islander** | 158 | 9409587 | 2.84 | 2.38 | 3.29 |
| **2007** | **NH Asian or Pacific Islander** | 176 | 9760888 | 2.92 | 2.48 | 3.35 |
| **2008** | **NH Asian or Pacific Islander** | 177 | 10102040 | 2.78 | 2.36 | 3.2 |
| **2009** | **NH Asian or Pacific Islander** | 209 | 10429408 | 3.15 | 2.71 | 3.58 |
| **2010** | **NH Asian or Pacific Islander** | 200 | 10670562 | 2.89 | 2.49 | 3.3 |
| **2011** | **NH Asian or Pacific Islander** | 226 | 11055808 | 2.99 | 2.6 | 3.39 |
| **2012** | **NH Asian or Pacific Islander** | 272 | 11510641 | 3.39 | 2.98 | 3.8 |
| **2013** | **NH Asian or Pacific Islander** | 296 | 11931284 | 3.38 | 2.99 | 3.78 |
| **2014** | **NH Asian or Pacific Islander** | 301 | 12503895 | 3.21 | 2.85 | 3.58 |
| **2015** | **NH Asian or Pacific Islander** | 330 | 13054155 | 3.27 | 2.91 | 3.62 |
| **2016** | **NH Asian or Pacific Islander** | 358 | 13344756 | 3.3 | 2.95 | 3.64 |
| **2017** | **NH Asian or Pacific Islander** | 366 | 13938546 | 3.23 | 2.89 | 3.56 |
| **2018** | **NH Asian or Pacific Islander** | 418 | 14258765 | 3.49 | 3.15 | 3.83 |
| **2019** | **NH Asian or Pacific Islander** | 499 | 14536839 | 3.9 | 3.56 | 4.25 |
| **1999** | **NH Black or African American** | 459 | 19808077 | 3.31 | 3.01 | 3.62 |
| **2000** | **NH Black or African American** | 454 | 20058273 | 3.23 | 2.93 | 3.53 |
| **2001** | **NH Black or African American** | 494 | 20404945 | 3.45 | 3.14 | 3.75 |
| **2002** | **NH Black or African American** | 488 | 20695836 | 3.34 | 3.04 | 3.64 |
| **2003** | **NH Black or African American** | 520 | 20982115 | 3.51 | 3.2 | 3.81 |
| **2004** | **NH Black or African American** | 564 | 21327519 | 3.71 | 3.4 | 4.02 |
| **2005** | **NH Black or African American** | 564 | 21703691 | 3.67 | 3.36 | 3.98 |
| **2006** | **NH Black or African American** | 616 | 22092072 | 3.87 | 3.56 | 4.18 |
| **2007** | **NH Black or African American** | 638 | 22474128 | 3.93 | 3.62 | 4.24 |
| **2008** | **NH Black or African American** | 662 | 22857307 | 3.96 | 3.66 | 4.27 |
| **2009** | **NH Black or African American** | 675 | 23236715 | 3.94 | 3.63 | 4.24 |
| **2010** | **NH Black or African American** | 776 | 23537629 | 4.37 | 4.06 | 4.69 |
| **2011** | **NH Black or African American** | 844 | 23958352 | 4.73 | 4.4 | 5.05 |
| **2012** | **NH Black or African American** | 951 | 24345607 | 5 | 4.68 | 5.33 |
| **2013** | **NH Black or African American** | 1027 | 24743381 | 5.21 | 4.88 | 5.53 |
| **2014** | **NH Black or African American** | 1022 | 25244585 | 4.95 | 4.64 | 5.26 |
| **2015** | **NH Black or African American** | 1070 | 25752287 | 5.05 | 4.74 | 5.36 |
| **2016** | **NH Black or African American** | 1224 | 26212105 | 5.64 | 5.31 | 5.96 |
| **2017** | **NH Black or African American** | 1269 | 26762620 | 5.54 | 5.23 | 5.86 |
| **2018** | **NH Black or African American** | 1379 | 27178229 | 5.82 | 5.51 | 6.14 |
| **2019** | **NH Black or African American** | 1509 | 27592123 | 6.13 | 5.81 | 6.44 |
| **1999** | **NH White** | 8032 | 134935890 | 5.32 | 5.21 | 5.44 |
| **2000** | **NH White** | 8611 | 135202971 | 5.64 | 5.52 | 5.76 |
| **2001** | **NH White** | 8875 | 135651679 | 5.77 | 5.65 | 5.89 |
| **2002** | **NH White** | 9384 | 136026843 | 6.03 | 5.9 | 6.15 |
| **2003** | **NH White** | 9731 | 136415868 | 6.18 | 6.06 | 6.3 |
| **2004** | **NH White** | 10033 | 136956601 | 6.31 | 6.19 | 6.43 |
| **2005** | **NH White** | 10578 | 137614452 | 6.54 | 6.42 | 6.67 |
| **2006** | **NH White** | 11205 | 138363022 | 6.85 | 6.73 | 6.98 |
| **2007** | **NH White** | 11753 | 139060265 | 7.09 | 6.96 | 7.22 |
| **2008** | **NH White** | 12003 | 139772453 | 7.14 | 7.01 | 7.27 |
| **2009** | **NH White** | 12406 | 140451863 | 7.29 | 7.16 | 7.42 |
| **2010** | **NH White** | 13212 | 140987505 | 7.64 | 7.51 | 7.77 |
| **2011** | **NH White** | 14146 | 141789725 | 8.03 | 7.9 | 8.16 |
| **2012** | **NH White** | 14937 | 142424668 | 8.32 | 8.18 | 8.45 |
| **2013** | **NH White** | 15414 | 143045234 | 8.44 | 8.31 | 8.58 |
| **2014** | **NH White** | 16275 | 143642265 | 8.77 | 8.63 | 8.91 |
| **2015** | **NH White** | 17320 | 144347874 | 9.19 | 9.05 | 9.33 |
| **2016** | **NH White** | 18390 | 144979180 | 9.58 | 9.44 | 9.72 |
| **2017** | **NH White** | 19892 | 145456222 | 10.18 | 10.04 | 10.33 |
| **2018** | **NH White** | 21524 | 145801376 | 10.76 | 10.61 | 10.9 |
| **2019** | **NH White** | 23327 | 146079678 | 11.44 | 11.29 | 11.59 |
| **1999** | **Hispanic or Latino** | 120 | 17503631 | 1.58 | 1.29 | 1.88 |
| **2000** | **Hispanic or Latino** | 180 | 18219679 | 2.29 | 1.95 | 2.64 |
| **2001** | **Hispanic or Latino** | 162 | 19290018 | 1.98 | 1.67 | 2.29 |
| **2002** | **Hispanic or Latino** | 187 | 20159630 | 2.16 | 1.84 | 2.48 |
| **2003** | **Hispanic or Latino** | 190 | 21011656 | 2 | 1.71 | 2.29 |
| **2004** | **Hispanic or Latino** | 212 | 21877214 | 2.2 | 1.89 | 2.5 |
| **2005** | **Hispanic or Latino** | 232 | 22804023 | 2.3 | 2 | 2.6 |
| **2006** | **Hispanic or Latino** | 261 | 23743864 | 2.48 | 2.17 | 2.78 |
| **2007** | **Hispanic or Latino** | 262 | 24673919 | 2.26 | 1.97 | 2.54 |
| **2008** | **Hispanic or Latino** | 285 | 25602850 | 2.37 | 2.09 | 2.65 |
| **2009** | **Hispanic or Latino** | 322 | 26504021 | 2.55 | 2.27 | 2.83 |
| **2010** | **Hispanic or Latino** | 394 | 27192663 | 2.96 | 2.66 | 3.26 |
| **2011** | **Hispanic or Latino** | 478 | 28255675 | 3.28 | 2.98 | 3.58 |
| **2012** | **Hispanic or Latino** | 485 | 28988437 | 3.16 | 2.88 | 3.45 |
| **2013** | **Hispanic or Latino** | 503 | 29784174 | 3.14 | 2.86 | 3.42 |
| **2014** | **Hispanic or Latino** | 587 | 30809714 | 3.38 | 3.1 | 3.66 |
| **2015** | **Hispanic or Latino** | 638 | 31761872 | 3.44 | 3.17 | 3.72 |
| **2016** | **Hispanic or Latino** | 752 | 32438262 | 3.87 | 3.59 | 4.16 |
| **2017** | **Hispanic or Latino** | 813 | 33594503 | 3.9 | 3.62 | 4.17 |
| **2018** | **Hispanic or Latino** | 933 | 34350362 | 4.3 | 4.02 | 4.58 |
| **2019** | **Hispanic or Latino** | 945 | 35025850 | 4.23 | 3.95 | 4.5 |

**Supplementary Table 3.** Cancer and AF–related age-adjusted mortality rates per 100,000, stratified by state in adults in the United States, 1999 to 2019.

| **State** | **Deaths** | **Population** | **Age Adjusted Rate** | **Age Adjusted Rate Lower 95% Confidence Interval** | **Age Adjusted Rate Upper 95% Confidence Interval** |
| --- | --- | --- | --- | --- | --- |
| **Alabama** | 3771 | 65528742 | 5.4 | 5.2 | 5.6 |
| **Alaska** | 436 | 9138485 | 8.4 | 7.6 | 9.3 |
| **Arizona** | 4002 | 85015692 | 4.3 | 4.2 | 4.4 |
| **Arkansas** | 2442 | 39659244 | 5.5 | 5.3 | 5.7 |
| **California** | 40151 | 503653297 | 8.4 | 8.3 | 8.5 |
| **Colorado** | 4998 | 69158969 | 8.5 | 8.3 | 8.7 |
| **Connecticut** | 5024 | 50512590 | 8.4 | 8.2 | 8.6 |
| **Delaware** | 931 | 12511368 | 6.8 | 6.4 | 7.3 |
| **District of Columbia** | 415 | 8937422 | 5.2 | 4.7 | 5.7 |
| **Florida** | 16847 | 273960905 | 4.7 | 4.6 | 4.7 |
| **Georgia** | 4594 | 128225447 | 4.3 | 4.1 | 4.4 |
| **Hawaii** | 1309 | 19071277 | 5.9 | 5.6 | 6.2 |
| **Idaho** | 1553 | 20183946 | 7.5 | 7.2 | 7.9 |
| **Illinois** | 10707 | 175818186 | 5.8 | 5.7 | 6 |
| **Indiana** | 6900 | 87854438 | 7.4 | 7.2 | 7.6 |
| **Iowa** | 4293 | 41906058 | 8 | 7.7 | 8.2 |
| **Kansas** | 2478 | 38006253 | 5.6 | 5.4 | 5.8 |
| **Kentucky** | 5179 | 59998174 | 8.4 | 8.2 | 8.6 |
| **Louisiana** | 2383 | 61747819 | 3.9 | 3.7 | 4.1 |
| **Maine** | 1973 | 19525435 | 8.5 | 8.1 | 8.8 |
| **Maryland** | 6693 | 80701083 | 8.6 | 8.4 | 8.8 |
| **Massachusetts** | 7545 | 94228617 | 7.1 | 6.9 | 7.2 |
| **Michigan** | 9622 | 138638733 | 6.4 | 6.2 | 6.5 |
| **Minnesota** | 8861 | 73140154 | 11.1 | 10.9 | 11.3 |
| **Mississippi** | 2580 | 39502729 | 6.3 | 6.1 | 6.6 |
| **Missouri** | 5885 | 82295948 | 6.3 | 6.2 | 6.5 |
| **Montana** | 1054 | 13824728 | 6.6 | 6.2 | 7 |
| **Nebraska** | 3119 | 24595597 | 10.7 | 10.3 | 11.1 |
| **Nevada** | 1093 | 36392390 | 3.4 | 3.2 | 3.6 |
| **New Hampshire** | 1958 | 18865744 | 9.9 | 9.5 | 10.4 |
| **New Jersey** | 10427 | 124744868 | 7.7 | 7.5 | 7.8 |
| **New Mexico** | 1204 | 27130846 | 4.3 | 4 | 4.5 |
| **New York** | 16206 | 274997468 | 5.4 | 5.3 | 5.5 |
| **North Carolina** | 9630 | 129934279 | 7.4 | 7.3 | 7.6 |
| **North Dakota** | 1206 | 9321511 | 10.1 | 9.5 | 10.7 |
| **Ohio** | 17767 | 161574318 | 9.8 | 9.6 | 9.9 |
| **Oklahoma** | 4580 | 50464843 | 8.4 | 8.2 | 8.7 |
| **Oregon** | 6366 | 54194105 | 10.7 | 10.4 | 10.9 |
| **Pennsylvania** | 19848 | 180935222 | 8.7 | 8.6 | 8.8 |
| **Rhode Island** | 2053 | 15017204 | 11.1 | 10.6 | 11.6 |
| **South Carolina** | 4593 | 63595863 | 7 | 6.8 | 7.2 |
| **South Dakota** | 1115 | 11012025 | 8 | 7.5 | 8.5 |
| **Tennessee** | 6747 | 87927940 | 7.5 | 7.3 | 7.7 |
| **Texas** | 20787 | 325545390 | 7.5 | 7.4 | 7.6 |
| **Utah** | 1345 | 32104656 | 5.1 | 4.8 | 5.4 |
| **Vermont** | 1203 | 8948072 | 11.9 | 11.3 | 12.6 |
| **Virginia** | 6338 | 110655176 | 6.1 | 5.9 | 6.2 |
| **Washington** | 9290 | 93561412 | 10.2 | 9.9 | 10.4 |
| **West Virginia** | 3158 | 26747989 | 9.9 | 9.6 | 10.3 |
| **Wisconsin** | 6295 | 78682411 | 7 | 6.8 | 7.2 |
| **Wyoming** | 526 | 7524408 | 7 | 6.4 | 7.6 |
|  | 319480 | 4247219476 | 7.1 | 7.1 | 7.2 |

**Supplementary Table 4.** Cancer and AF–related age-adjusted mortality rates per 100,000, stratified by census region in adults in the United States, 1999 to 2019.

| **Year** | **Census Region** | **Deaths** | **Population** | **Age Adjusted Rate** | **Age Adjusted Rate Lower 95% Confidence Interval** | **Age Adjusted Rate Upper 95% Confidence Interval** |
| --- | --- | --- | --- | --- | --- | --- |
| 1999 | **Northeast** | 2217 | 35633134 | 5.86 | 5.62 | 6.11 |
| 2000 | **Northeast** | 2374 | 35788687 | 6.2 | 5.95 | 6.45 |
| 2001 | **Northeast** | 2381 | 36006250 | 6.15 | 5.91 | 6.4 |
| 2002 | **Northeast** | 2453 | 36185082 | 6.27 | 6.02 | 6.52 |
| 2003 | **Northeast** | 2408 | 36346948 | 6.08 | 5.84 | 6.33 |
| 2004 | **Northeast** | 2566 | 36462699 | 6.45 | 6.2 | 6.7 |
| 2005 | **Northeast** | 2588 | 36559788 | 6.4 | 6.15 | 6.65 |
| 2006 | **Northeast** | 2760 | 36682176 | 6.78 | 6.52 | 7.03 |
| 2007 | **Northeast** | 2762 | 36846338 | 6.67 | 6.42 | 6.92 |
| 2008 | **Northeast** | 2794 | 37084149 | 6.69 | 6.44 | 6.94 |
| 2009 | **Northeast** | 2856 | 37339597 | 6.75 | 6.5 | 7 |
| 2010 | **Northeast** | 3170 | 37543347 | 7.39 | 7.13 | 7.65 |
| 2011 | **Northeast** | 3419 | 37864117 | 7.88 | 7.61 | 8.15 |
| 2012 | **Northeast** | 3525 | 38158527 | 7.96 | 7.7 | 8.23 |
| 2013 | **Northeast** | 3610 | 38437194 | 7.99 | 7.72 | 8.25 |
| 2014 | **Northeast** | 3636 | 38710627 | 7.96 | 7.7 | 8.22 |
| 2015 | **Northeast** | 3820 | 38965872 | 8.22 | 7.96 | 8.49 |
| 2016 | **Northeast** | 3920 | 39040202 | 8.37 | 8.11 | 8.64 |
| 2017 | **Northeast** | 4165 | 39417175 | 8.62 | 8.35 | 8.88 |
| 2018 | **Northeast** | 4342 | 39321978 | 8.84 | 8.57 | 9.11 |
| 2019 | **Northeast** | 4471 | 39381333 | 8.94 | 8.67 | 9.2 |
| 1999 | **Midwest** | 2236 | 41293967 | 5.24 | 5.02 | 5.45 |
| 2000 | **Midwest** | 2404 | 41504992 | 5.6 | 5.38 | 5.82 |
| 2001 | **Midwest** | 2472 | 41762990 | 5.68 | 5.45 | 5.9 |
| 2002 | **Midwest** | 2647 | 41970731 | 6.02 | 5.79 | 6.25 |
| 2003 | **Midwest** | 2746 | 42200881 | 6.19 | 5.96 | 6.42 |
| 2004 | **Midwest** | 2853 | 42455980 | 6.36 | 6.12 | 6.59 |
| 2005 | **Midwest** | 3046 | 42748579 | 6.72 | 6.48 | 6.96 |
| 2006 | **Midwest** | 3197 | 43084311 | 6.93 | 6.69 | 7.17 |
| 2007 | **Midwest** | 3311 | 43424366 | 7.08 | 6.83 | 7.32 |
| 2008 | **Midwest** | 3438 | 43718509 | 7.23 | 6.99 | 7.47 |
| 2009 | **Midwest** | 3560 | 44010460 | 7.43 | 7.18 | 7.67 |
| 2010 | **Midwest** | 3702 | 44248465 | 7.64 | 7.39 | 7.89 |
| 2011 | **Midwest** | 3858 | 44584105 | 7.82 | 7.57 | 8.07 |
| 2012 | **Midwest** | 3872 | 44817227 | 7.68 | 7.44 | 7.93 |
| 2013 | **Midwest** | 4040 | 45090597 | 7.89 | 7.64 | 8.13 |
| 2014 | **Midwest** | 4355 | 45360409 | 8.38 | 8.13 | 8.64 |
| 2015 | **Midwest** | 4683 | 45628315 | 8.87 | 8.61 | 9.12 |
| 2016 | **Midwest** | 4894 | 45802491 | 9.1 | 8.85 | 9.36 |
| 2017 | **Midwest** | 5228 | 46143783 | 9.51 | 9.25 | 9.77 |
| 2018 | **Midwest** | 5586 | 46405110 | 9.95 | 9.69 | 10.22 |
| 2019 | **Midwest** | 6120 | 46589364 | 10.7 | 10.43 | 10.97 |
| 1999 | **South** | 2522 | 64108630 | 4.11 | 3.95 | 4.27 |
| 2000 | **South** | 2689 | 64843390 | 4.34 | 4.17 | 4.5 |
| 2001 | **South** | 2807 | 65874012 | 4.47 | 4.3 | 4.63 |
| 2002 | **South** | 2986 | 66758178 | 4.69 | 4.52 | 4.85 |
| 2003 | **South** | 3107 | 67639133 | 4.79 | 4.62 | 4.96 |
| 2004 | **South** | 3154 | 68718205 | 4.79 | 4.62 | 4.96 |
| 2005 | **South** | 3341 | 69951038 | 4.95 | 4.78 | 5.12 |
| 2006 | **South** | 3575 | 71198336 | 5.18 | 5.01 | 5.35 |
| 2007 | **South** | 3878 | 72387467 | 5.5 | 5.33 | 5.68 |
| 2008 | **South** | 3895 | 73529724 | 5.4 | 5.23 | 5.57 |
| 2009 | **South** | 4214 | 74596130 | 5.72 | 5.55 | 5.89 |
| 2010 | **South** | 4462 | 75419767 | 5.93 | 5.75 | 6.1 |
| 2011 | **South** | 4782 | 76650163 | 6.14 | 5.96 | 6.31 |
| 2012 | **South** | 5376 | 77681893 | 6.69 | 6.51 | 6.87 |
| 2013 | **South** | 5589 | 78693557 | 6.76 | 6.58 | 6.94 |
| 2014 | **South** | 5919 | 79945474 | 6.91 | 6.73 | 7.09 |
| 2015 | **South** | 6335 | 81260812 | 7.21 | 7.03 | 7.39 |
| 2016 | **South** | 6966 | 82405493 | 7.7 | 7.51 | 7.88 |
| 2017 | **South** | 7770 | 83659214 | 8.36 | 8.17 | 8.55 |
| 2018 | **South** | 8737 | 84717024 | 9.08 | 8.89 | 9.27 |
| 2019 | **South** | 9564 | 85606773 | 9.7 | 9.51 | 9.9 |
| 1999 | **West** | 1746 | 39373038 | 5.09 | 4.85 | 5.33 |
| 2000 | **West** | 1908 | 39847571 | 5.46 | 5.21 | 5.7 |
| 2001 | **West** | 2001 | 40661876 | 5.59 | 5.35 | 5.84 |
| 2002 | **West** | 2098 | 41294037 | 5.72 | 5.48 | 5.97 |
| 2003 | **West** | 2338 | 41903467 | 6.26 | 6.01 | 6.52 |
| 2004 | **West** | 2404 | 42568500 | 6.29 | 6.04 | 6.54 |
| 2005 | **West** | 2583 | 43291979 | 6.59 | 6.33 | 6.84 |
| 2006 | **West** | 2758 | 44054536 | 6.85 | 6.59 | 7.11 |
| 2007 | **West** | 2923 | 44745606 | 7.09 | 6.83 | 7.35 |
| 2008 | **West** | 3038 | 45462708 | 7.18 | 6.93 | 7.44 |
| 2009 | **West** | 3046 | 46160829 | 7.01 | 6.76 | 7.26 |
| 2010 | **West** | 3317 | 46680404 | 7.46 | 7.21 | 7.72 |
| 2011 | **West** | 3694 | 47494551 | 8.01 | 7.75 | 8.27 |
| 2012 | **West** | 3939 | 48168390 | 8.28 | 8.02 | 8.54 |
| 2013 | **West** | 4096 | 48863966 | 8.33 | 8.07 | 8.59 |
| 2014 | **West** | 4399 | 49792770 | 8.7 | 8.44 | 8.96 |
| 2015 | **West** | 4652 | 50698818 | 8.87 | 8.61 | 9.13 |
| 2016 | **West** | 5069 | 51393231 | 9.4 | 9.14 | 9.66 |
| 2017 | **West** | 5306 | 52227159 | 9.64 | 9.38 | 9.9 |
| 2018 | **West** | 5734 | 52867078 | 10.13 | 9.86 | 10.39 |
| 2019 | **West** | 6278 | 53403697 | 10.72 | 10.46 | 10.99 |

**Supplementary Table 5.** Cancer and AF–related age-adjusted mortality rates per 100,000, stratified by urbanization in adults in the United States, 1999 to 2019.

| **Year** | **Urbanization** | **Deaths** | **Population** | **Age Adjusted Rate** | **Age Adjusted Rate Lower 95% Confidence Interval** | **Age Adjusted Rate Upper 95% Confidence Interval** |
| --- | --- | --- | --- | --- | --- | --- |
| **1999** | **Metropolitan** | 6998 | 151245342 | 4.92 | 4.81 | 5.04 |
| **2000** | **Metropolitan** | 7498 | 152658699 | 5.2 | 5.09 | 5.32 |
| **2001** | **Metropolitan** | 7691 | 154896258 | 5.25 | 5.13 | 5.37 |
| **2002** | **Metropolitan** | 8089 | 156659051 | 5.45 | 5.33 | 5.56 |
| **2003** | **Metropolitan** | 8505 | 158367715 | 5.63 | 5.51 | 5.75 |
| **2004** | **Metropolitan** | 8723 | 160272097 | 5.68 | 5.57 | 5.8 |
| **2005** | **Metropolitan** | 9341 | 162371826 | 5.96 | 5.84 | 6.08 |
| **2006** | **Metropolitan** | 9910 | 164523389 | 6.19 | 6.07 | 6.31 |
| **2007** | **Metropolitan** | 10224 | 166650886 | 6.27 | 6.15 | 6.4 |
| **2008** | **Metropolitan** | 10486 | 168826027 | 6.3 | 6.18 | 6.42 |
| **2009** | **Metropolitan** | 10920 | 170965574 | 6.42 | 6.3 | 6.54 |
| **2010** | **Metropolitan** | 11758 | 172591105 | 6.79 | 6.67 | 6.92 |
| **2011** | **Metropolitan** | 12821 | 175204532 | 7.2 | 7.08 | 7.33 |
| **2012** | **Metropolitan** | 13509 | 177423676 | 7.38 | 7.25 | 7.5 |
| **2013** | **Metropolitan** | 14047 | 179634449 | 7.49 | 7.36 | 7.61 |
| **2014** | **Metropolitan** | 14738 | 182304016 | 7.65 | 7.53 | 7.78 |
| **2015** | **Metropolitan** | 15638 | 184959306 | 7.91 | 7.78 | 8.03 |
| **2016** | **Metropolitan** | 16734 | 186963190 | 8.24 | 8.11 | 8.36 |
| **2017** | **Metropolitan** | 18123 | 189675062 | 8.71 | 8.59 | 8.84 |
| **2018** | **Metropolitan** | 19476 | 191415024 | 9.11 | 8.98 | 9.24 |
| **2019** | **Metropolitan** | 21006 | 193006488 | 9.58 | 9.45 | 9.71 |
| **1999** | **Nonmetropolitan** | 1723 | 29163427 | 5.05 | 4.81 | 5.28 |
| **2000** | **Nonmetropolitan** | 1877 | 29325941 | 5.47 | 5.22 | 5.71 |
| **2001** | **Nonmetropolitan** | 1970 | 29408870 | 5.69 | 5.44 | 5.94 |
| **2002** | **Nonmetropolitan** | 2095 | 29548977 | 6.03 | 5.77 | 6.29 |
| **2003** | **Nonmetropolitan** | 2094 | 29722714 | 5.98 | 5.72 | 6.24 |
| **2004** | **Nonmetropolitan** | 2254 | 29933287 | 6.4 | 6.13 | 6.66 |
| **2005** | **Nonmetropolitan** | 2217 | 30179558 | 6.22 | 5.96 | 6.48 |
| **2006** | **Nonmetropolitan** | 2380 | 30495970 | 6.59 | 6.32 | 6.86 |
| **2007** | **Nonmetropolitan** | 2650 | 30752891 | 7.25 | 6.98 | 7.53 |
| **2008** | **Nonmetropolitan** | 2679 | 30969063 | 7.2 | 6.92 | 7.47 |
| **2009** | **Nonmetropolitan** | 2756 | 31141442 | 7.37 | 7.09 | 7.64 |
| **2010** | **Nonmetropolitan** | 2893 | 31300878 | 7.64 | 7.36 | 7.92 |
| **2011** | **Nonmetropolitan** | 2932 | 31388404 | 7.58 | 7.3 | 7.86 |
| **2012** | **Nonmetropolitan** | 3203 | 31402361 | 8.17 | 7.89 | 8.46 |
| **2013** | **Nonmetropolitan** | 3288 | 31450865 | 8.22 | 7.94 | 8.51 |
| **2014** | **Nonmetropolitan** | 3571 | 31505264 | 8.79 | 8.5 | 9.08 |
| **2015** | **Nonmetropolitan** | 3852 | 31594511 | 9.32 | 9.02 | 9.61 |
| **2016** | **Nonmetropolitan** | 4115 | 31678227 | 9.81 | 9.51 | 10.11 |
| **2017** | **Nonmetropolitan** | 4346 | 31772269 | 10.16 | 9.86 | 10.46 |
| **2018** | **Nonmetropolitan** | 4923 | 31896166 | 11.3 | 10.98 | 11.62 |
| **2019** | **Nonmetropolitan** | 5427 | 31974679 | 12.18 | 11.86 | 12.51 |

**Supplementary Table 6.** Cancer related age-adjusted mortality rates per 100,000 adults in the United States, 1999 to 2019.

| **Year** | **Deaths** | **Population** | **Age Adjusted Rate** | **Age Adjusted Rate Lower 95% Confidence Interval** | **Age Adjusted Rate Upper 95% Confidence Interval** |
| --- | --- | --- | --- | --- | --- |
| **1999** | 608473 | 180408769 | 343.73 | 342.87 | 344.6 |
| **2000** | 610343 | 181984640 | 340.83 | 339.98 | 341.69 |
| **2001** | 610282 | 184305128 | 335.04 | 334.2 | 335.88 |
| **2002** | 613321 | 186208028 | 331.06 | 330.23 | 331.89 |
| **2003** | 612019 | 188090429 | 324.64 | 323.83 | 325.46 |
| **2004** | 607413 | 190205384 | 317.07 | 316.27 | 317.87 |
| **2005** | 613345 | 192551384 | 314.15 | 313.36 | 314.94 |
| **2006** | 613454 | 195019359 | 308.23 | 307.46 | 309 |
| **2007** | 615393 | 197403777 | 303.26 | 302.5 | 304.02 |
| **2008** | 618653 | 199795090 | 298.54 | 297.79 | 299.29 |
| **2009** | 619400 | 202107016 | 292.88 | 292.15 | 293.62 |
| **2010** | 627223 | 203891983 | 291.71 | 290.99 | 292.44 |
| **2011** | 628318 | 206592936 | 284.66 | 283.95 | 285.37 |
| **2012** | 635506 | 208826037 | 280.74 | 280.04 | 281.44 |
| **2013** | 637894 | 211085314 | 275.17 | 274.49 | 275.85 |
| **2014** | 644396 | 213809280 | 271.34 | 270.67 | 272.01 |
| **2015** | 649789 | 216553817 | 267.08 | 266.42 | 267.74 |
| **2016** | 653304 | 218641417 | 262.96 | 262.31 | 263.6 |
| **2017** | 657136 | 221447331 | 258.35 | 257.71 | 258.98 |
| **2018** | 659873 | 223311190 | 253.54 | 252.92 | 254.16 |
| **2019** | 662094 | 224981167 | 249.2 | 248.59 | 249.81 |
|  | 13197629 | 4247219476 | 291.87 | 291.71 | 292.02 |

**Supplementary Table 7.** AF related age-adjusted mortality rates per 100,000 adults in the United States, 1999 to 2019.

| **Year** | **Deaths** | **Population** | **Age Adjusted Rate** | **Age Adjusted Rate Lower 95% Confidence Interval** | **Age Adjusted Rate Upper 95% Confidence Interval** |
| --- | --- | --- | --- | --- | --- |
| **1999** | 66863 | 180408769 | 38.16 | 37.87 | 38.45 |
| **2000** | 70693 | 181984640 | 39.75 | 39.46 | 40.05 |
| **2001** | 73287 | 184305128 | 40.57 | 40.28 | 40.87 |
| **2002** | 77744 | 186208028 | 42.47 | 42.18 | 42.77 |
| **2003** | 80394 | 188090429 | 43.17 | 42.87 | 43.46 |
| **2004** | 80712 | 190205384 | 42.73 | 42.44 | 43.03 |
| **2005** | 87954 | 192551384 | 45.61 | 45.31 | 45.91 |
| **2006** | 89955 | 195019359 | 45.62 | 45.32 | 45.92 |
| **2007** | 93577 | 197403777 | 46.43 | 46.14 | 46.73 |
| **2008** | 99271 | 199795090 | 48.23 | 47.93 | 48.53 |
| **2009** | 100180 | 202107016 | 47.68 | 47.38 | 47.97 |
| **2010** | 107320 | 203891983 | 50.2 | 49.9 | 50.5 |
| **2011** | 116236 | 206592936 | 52.71 | 52.41 | 53.02 |
| **2012** | 123018 | 208826037 | 54.43 | 54.13 | 54.74 |
| **2013** | 131902 | 211085314 | 56.97 | 56.66 | 57.28 |
| **2014** | 137419 | 213809280 | 58.05 | 57.75 | 58.36 |
| **2015** | 148663 | 216553817 | 61.34 | 61.03 | 61.66 |
| **2016** | 154804 | 218641417 | 62.56 | 62.25 | 62.88 |
| **2017** | 166777 | 221447331 | 65.88 | 65.56 | 66.2 |
| **2018** | 175307 | 223311190 | 67.65 | 67.33 | 67.96 |
| **2019** | 183305 | 224981167 | 69.32 | 69 | 69.64 |
|  | 2365381 | 4247219476 | 52.62 | 52.55 | 52.69 |

**Supplementary Table 8.** Cancer and AF–related age-adjusted mortality rates per 100,000, stratified by cancer subtype in adults in the United States, 1999 to 2019

| **Year** | **Cancer Subtype** | **Deaths** | **Population** | **Age Adjusted Rate** | **Age Adjusted Rate Lower 95% Confidence Interval** | **Age Adjusted Rate Upper 95% Confidence Interval** |
| --- | --- | --- | --- | --- | --- | --- |
| **1999** | Lung Cancer | 1953 | 180000000 | 1.1 | 1.05 | 1.15 |
| **2000** | Lung Cancer | 2124 | 182000000 | 1.2 | 1.15 | 1.25 |
| **2001** | Lung Cancer | 2209 | 184000000 | 1.21 | 1.16 | 1.26 |
| **2002** | Lung Cancer | 2362 | 186000000 | 1.29 | 1.24 | 1.35 |
| **2003** | Lung Cancer | 2405 | 188000000 | 1.29 | 1.24 | 1.34 |
| **2004** | Lung Cancer | 2632 | 190000000 | 1.39 | 1.34 | 1.45 |
| **2005** | Lung Cancer | 2681 | 193000000 | 1.4 | 1.35 | 1.46 |
| **2006** | Lung Cancer | 2831 | 195000000 | 1.44 | 1.39 | 1.5 |
| **2007** | Lung Cancer | 3025 | 197000000 | 1.52 | 1.47 | 1.58 |
| **2008** | Lung Cancer | 3123 | 200000000 | 1.55 | 1.49 | 1.6 |
| **2009** | Lung Cancer | 3202 | 202000000 | 1.56 | 1.5 | 1.61 |
| **2010** | Lung Cancer | 3395 | 204000000 | 1.62 | 1.56 | 1.67 |
| **2011** | Lung Cancer | 3643 | 207000000 | 1.7 | 1.64 | 1.75 |
| **2012** | Lung Cancer | 4023 | 209000000 | 1.84 | 1.78 | 1.89 |
| **2013** | Lung Cancer | 4089 | 211000000 | 1.8 | 1.74 | 1.86 |
| **2014** | Lung Cancer | 4300 | 214000000 | 1.86 | 1.8 | 1.91 |
| **2015** | Lung Cancer | 4546 | 217000000 | 1.9 | 1.84 | 1.95 |
| **2016** | Lung Cancer | 4840 | 219000000 | 1.98 | 1.93 | 2.04 |
| **2017** | Lung Cancer | 5142 | 221000000 | 2.05 | 1.99 | 2.1 |
| **2018** | Lung Cancer | 5448 | 223000000 | 2.1 | 2.04 | 2.15 |
| **2019** | Lung Cancer | 5864 | 225000000 | 2.22 | 2.16 | 2.28 |
| **1999** | Gastrointestinal Cancer | 2013 | 180000000 | 1.15 | 1.1 | 1.2 |
| **2000** | Gastrointestinal Cancer | 2169 | 182000000 | 1.21 | 1.16 | 1.26 |
| **2001** | Gastrointestinal Cancer | 2229 | 184000000 | 1.22 | 1.17 | 1.27 |
| **2002** | Gastrointestinal Cancer | 2254 | 186000000 | 1.23 | 1.18 | 1.28 |
| **2003** | Gastrointestinal Cancer | 2413 | 188000000 | 1.29 | 1.24 | 1.35 |
| **2004** | Gastrointestinal Cancer | 2417 | 190000000 | 1.28 | 1.22 | 1.33 |
| **2005** | Gastrointestinal Cancer | 2592 | 193000000 | 1.33 | 1.28 | 1.38 |
| **2006** | Gastrointestinal Cancer | 2673 | 195000000 | 1.35 | 1.3 | 1.4 |
| **2007** | Gastrointestinal Cancer | 2714 | 197000000 | 1.34 | 1.29 | 1.39 |
| **2008** | Gastrointestinal Cancer | 2800 | 200000000 | 1.39 | 1.34 | 1.44 |
| **2009** | Gastrointestinal Cancer | 2875 | 202000000 | 1.38 | 1.33 | 1.43 |
| **2010** | Gastrointestinal Cancer | 3040 | 204000000 | 1.43 | 1.38 | 1.48 |
| **2011** | Gastrointestinal Cancer | 3347 | 207000000 | 1.55 | 1.5 | 1.6 |
| **2012** | Gastrointestinal Cancer | 3433 | 209000000 | 1.55 | 1.5 | 1.6 |
| **2013** | Gastrointestinal Cancer | 3636 | 211000000 | 1.6 | 1.55 | 1.66 |
| **2014** | Gastrointestinal Cancer | 3734 | 214000000 | 1.61 | 1.56 | 1.66 |
| **2015** | Gastrointestinal Cancer | 3901 | 217000000 | 1.62 | 1.57 | 1.67 |
| **2016** | Gastrointestinal Cancer | 4245 | 219000000 | 1.74 | 1.69 | 1.79 |
| **2017** | Gastrointestinal Cancer | 4658 | 221000000 | 1.84 | 1.79 | 1.89 |
| **2018** | Gastrointestinal Cancer | 4894 | 223000000 | 1.89 | 1.84 | 1.94 |
| **2019** | Gastrointestinal Cancer | 5354 | 225000000 | 2.03 | 1.97 | 2.08 |
| **1999** | Breast Cancer | 828 | 180000000 | 0.46 | 0.43 | 0.5 |
| **2000** | Breast Cancer | 890 | 182000000 | 0.5 | 0.47 | 0.53 |
| **2001** | Breast Cancer | 956 | 184000000 | 0.52 | 0.49 | 0.56 |
| **2002** | Breast Cancer | 1011 | 186000000 | 0.55 | 0.51 | 0.58 |
| **2003** | Breast Cancer | 1021 | 188000000 | 0.55 | 0.51 | 0.58 |
| **2004** | Breast Cancer | 1003 | 190000000 | 0.53 | 0.5 | 0.56 |
| **2005** | Breast Cancer | 1108 | 193000000 | 0.57 | 0.54 | 0.6 |
| **2006** | Breast Cancer | 1193 | 195000000 | 0.59 | 0.56 | 0.63 |
| **2007** | Breast Cancer | 1268 | 197000000 | 0.62 | 0.59 | 0.66 |
| **2008** | Breast Cancer | 1255 | 200000000 | 0.6 | 0.57 | 0.64 |
| **2009** | Breast Cancer | 1284 | 202000000 | 0.61 | 0.57 | 0.64 |
| **2010** | Breast Cancer | 1442 | 204000000 | 0.68 | 0.64 | 0.71 |
| **2011** | Breast Cancer | 1439 | 207000000 | 0.66 | 0.63 | 0.7 |
| **2012** | Breast Cancer | 1497 | 209000000 | 0.66 | 0.62 | 0.69 |
| **2013** | Breast Cancer | 1549 | 211000000 | 0.67 | 0.63 | 0.7 |
| **2014** | Breast Cancer | 1695 | 214000000 | 0.72 | 0.68 | 0.75 |
| **2015** | Breast Cancer | 1752 | 217000000 | 0.72 | 0.69 | 0.75 |
| **2016** | Breast Cancer | 1785 | 219000000 | 0.72 | 0.68 | 0.75 |
| **2017** | Breast Cancer | 1983 | 221000000 | 0.78 | 0.75 | 0.82 |
| **2018** | Breast Cancer | 2144 | 223000000 | 0.82 | 0.78 | 0.85 |
| **2019** | Breast Cancer | 2310 | 225000000 | 0.87 | 0.83 | 0.91 |
| **1999** | Prostate Cancer | 1211 | 180000000 | 0.68 | 0.64 | 0.71 |
| **2000** | Prostate Cancer | 1284 | 182000000 | 0.71 | 0.67 | 0.75 |
| **2001** | Prostate Cancer | 1328 | 184000000 | 0.74 | 0.7 | 0.78 |
| **2002** | Prostate Cancer | 1343 | 186000000 | 0.74 | 0.7 | 0.77 |
| **2003** | Prostate Cancer | 1409 | 188000000 | 0.75 | 0.71 | 0.79 |
| **2004** | Prostate Cancer | 1457 | 190000000 | 0.77 | 0.73 | 0.81 |
| **2005** | Prostate Cancer | 1510 | 193000000 | 0.8 | 0.76 | 0.84 |
| **2006** | Prostate Cancer | 1653 | 195000000 | 0.85 | 0.81 | 0.89 |
| **2007** | Prostate Cancer | 1674 | 197000000 | 0.84 | 0.8 | 0.88 |
| **2008** | Prostate Cancer | 1708 | 200000000 | 0.84 | 0.8 | 0.88 |
| **2009** | Prostate Cancer | 1771 | 202000000 | 0.86 | 0.82 | 0.9 |
| **2010** | Prostate Cancer | 1859 | 204000000 | 0.88 | 0.84 | 0.92 |
| **2011** | Prostate Cancer | 2005 | 207000000 | 0.92 | 0.88 | 0.96 |
| **2012** | Prostate Cancer | 1977 | 209000000 | 0.88 | 0.84 | 0.92 |
| **2013** | Prostate Cancer | 2066 | 211000000 | 0.91 | 0.87 | 0.95 |
| **2014** | Prostate Cancer | 2169 | 214000000 | 0.93 | 0.89 | 0.97 |
| **2015** | Prostate Cancer | 2267 | 217000000 | 0.95 | 0.91 | 0.99 |
| **2016** | Prostate Cancer | 2432 | 219000000 | 1 | 0.96 | 1.04 |
| **2017** | Prostate Cancer | 2588 | 221000000 | 1.04 | 1 | 1.08 |
| **2018** | Prostate Cancer | 2925 | 223000000 | 1.13 | 1.09 | 1.17 |
| **2019** | Prostate Cancer | 3190 | 225000000 | 1.21 | 1.16 | 1.25 |
| **1999** | Hematologic Cancer | 1081 | 180000000 | 0.61 | 0.57 | 0.65 |
| **2000** | Hematologic Cancer | 1156 | 182000000 | 0.64 | 0.6 | 0.68 |
| **2001** | Hematologic Cancer | 1168 | 184000000 | 0.64 | 0.61 | 0.68 |
| **2002** | Hematologic Cancer | 1339 | 186000000 | 0.72 | 0.69 | 0.76 |
| **2003** | Hematologic Cancer | 1368 | 188000000 | 0.72 | 0.68 | 0.76 |
| **2004** | Hematologic Cancer | 1398 | 190000000 | 0.72 | 0.69 | 0.76 |
| **2005** | Hematologic Cancer | 1426 | 193000000 | 0.73 | 0.69 | 0.77 |
| **2006** | Hematologic Cancer | 1593 | 195000000 | 0.8 | 0.76 | 0.84 |
| **2007** | Hematologic Cancer | 1752 | 197000000 | 0.87 | 0.83 | 0.91 |
| **2008** | Hematologic Cancer | 1791 | 200000000 | 0.88 | 0.84 | 0.92 |
| **2009** | Hematologic Cancer | 1902 | 202000000 | 0.91 | 0.87 | 0.95 |
| **2010** | Hematologic Cancer | 2102 | 204000000 | 1 | 0.95 | 1.04 |
| **2011** | Hematologic Cancer | 2232 | 207000000 | 1.05 | 1 | 1.09 |
| **2012** | Hematologic Cancer | 2450 | 209000000 | 1.1 | 1.06 | 1.14 |
| **2013** | Hematologic Cancer | 2469 | 211000000 | 1.07 | 1.03 | 1.11 |
| **2014** | Hematologic Cancer | 2712 | 214000000 | 1.17 | 1.12 | 1.21 |
| **2015** | Hematologic Cancer | 2910 | 217000000 | 1.22 | 1.18 | 1.27 |
| **2016** | Hematologic Cancer | 3186 | 219000000 | 1.32 | 1.27 | 1.37 |
| **2017** | Hematologic Cancer | 3402 | 221000000 | 1.38 | 1.33 | 1.43 |
| **2018** | Hematologic Cancer | 3813 | 223000000 | 1.49 | 1.44 | 1.54 |
| **2019** | Hematologic Cancer | 4067 | 225000000 | 1.55 | 1.5 | 1.59 |

**Supplementary Figure 1.** Atrial fibrillation–related and Cancer–related age-adjusted mortality rates per 100,000 adults in the United States, 1999 to 2019.

**
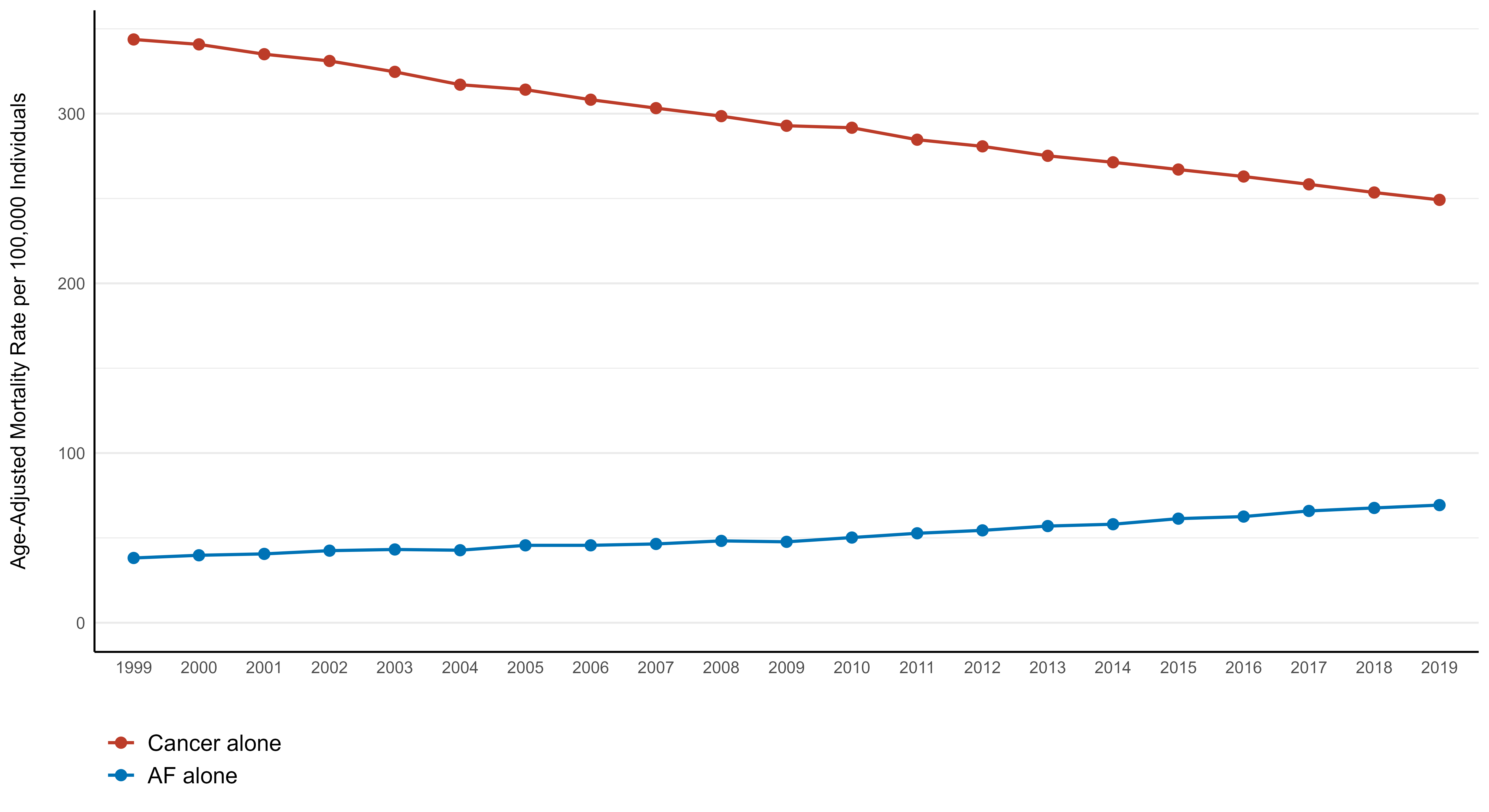
**

**Supplementary Figure 2.** Cancer and AF–related age-adjusted mortality rates per 100,000, stratified by cancer subtype in adults in the United States, 1999 to 2019.
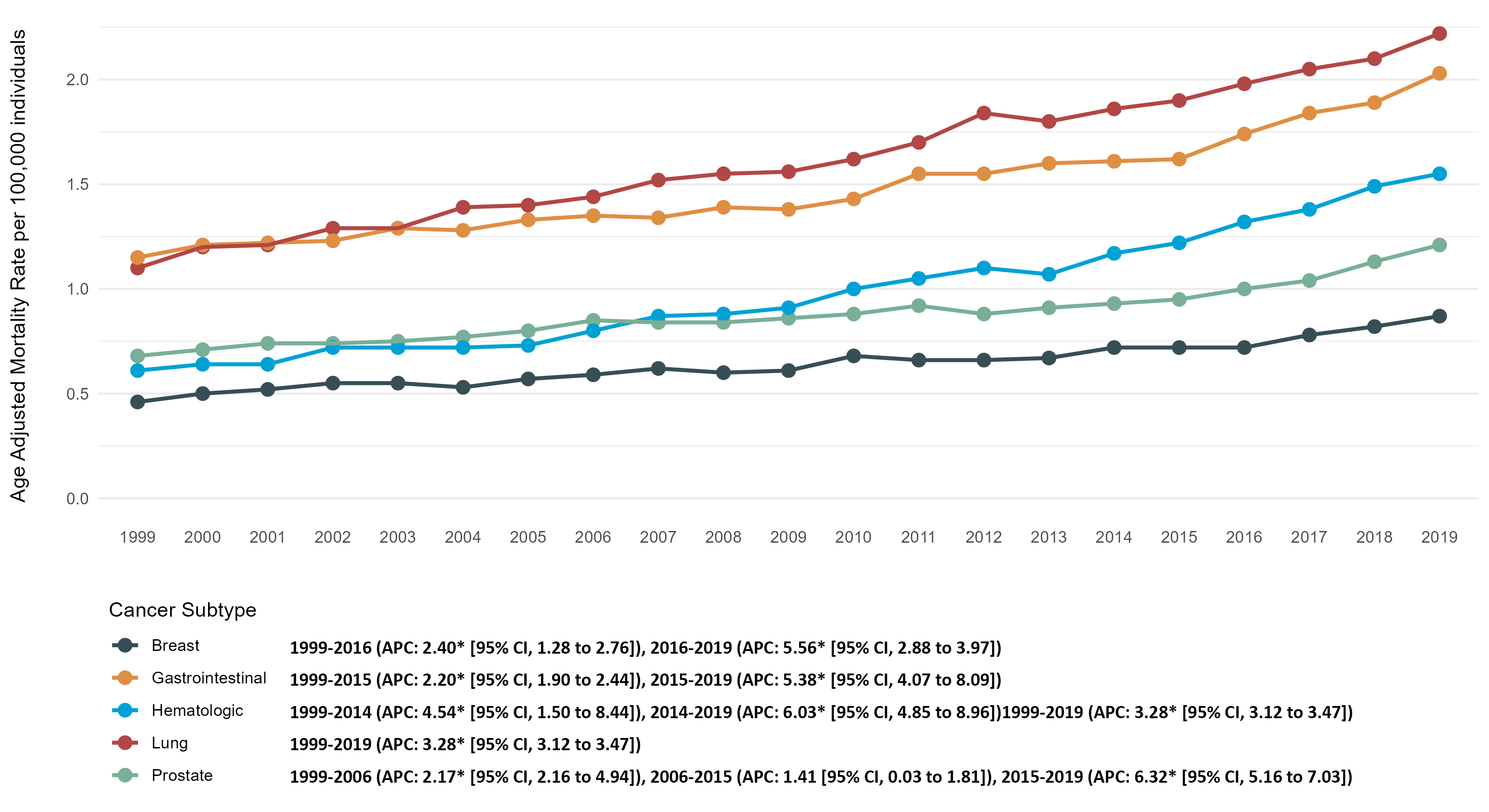

Supplement: Supplementary Tables 1-8 and Supplementary Figures 1 and 2 [file mmc1.docx]
